# Factors impacting physicians’ decisions to prevent variceal hemorrhage

**DOI:** 10.1186/s12876-015-0287-1

**Published:** 2015-05-02

**Authors:** Kathleen Yan, John FP Bridges, Salvador Augustin, Loren Laine, Guadalupe Garcia-Tsao, Liana Fraenkel

**Affiliations:** 1 Section of Rheumatology, Yale University School of Medicine, 300 Cedar ST, TAC Bldg, RM #525, P.O. Box 208031, New Haven, CT USA; 2 Yale University School of Medicine, New Haven, Connecticut and VA Connecticut Health Care System, West Haven, CT USA; 3 Department of Health Policy and Management, John Hopkins Bloomberg School of Public Health, Baltimore, MD USA

**Keywords:** Variceal hemorrhage, Treatment preferences, Discrete choice, Best worst scaling

## Abstract

**Background:**

Reasons underlying the variability of physicians’ preferences for non-selective beta-blockers (BBs) and endoscopic variceal ligation (EVL) to prevent a first variceal bleed have not been empirically studied. Our aims were to examine whether 1) gastroenterologists can be classified into distinct subgroups based on how they prioritize specific treatment attributes and 2) physician characteristics are associated with treatment preference.

**Methods:**

We surveyed physicians to determine their preferred treatment for a standardized patient with large varices and examined the influence of treatment characteristics on physicians’ decision making using best-worst scaling. Latent class analysis was used to examine whether physicians could be classified into groups with similar decision-making styles.

**Results:**

110 physicians were interviewed (participation rate 39%). The majority spent two or more days a week performing endoscopies and had practices comprising less than 25% of patients with liver disease. Latent class analysis demonstrated that physicians could be classified into at least two distinct groups. Most (n = 80, Group 1) were influenced solely by the ability to visually confirm eradication of varices. In contrast, members of Group 2 (n = 30) were influenced by the side effects and mechanism of action of BBs. Group 1 members were more likely to have practices that included fewer patients with liver disease and more likely to choose options including EVL (p = 0.01 for both).

**Conclusions:**

Among physicians, where the majority performs endoscopy on two or more days per week, most prefer prevention strategies which include EVL. This may be due to the strong appeal of being able to visualize eradication of varices.

## Background

Gastroesophageal varices are a common complication of portal hypertension, developing in approximately 50% of patients with cirrhosis [1]. The risk of bleeding in patients with moderate to large varices (where moderate refers to varices elevated above the mucosal surface but occupying less than one-third of the esophageal lumen and large occupy more than one-third of the esophageal lumen) is up to 15% per year and variceal hemorrhage is associated with a mortality rate of 20% at six weeks [2]. Given the high prevalence of gastroesophageal varices among patients with cirrhosis, and the high risk of mortality associated with bleeding, screening for varices and use of prophylactic therapies to prevent a first variceal hemorrhage are critical components of care.

Two treatment options have been proven to be effective in preventing first variceal bleed in patients with cirrhosis and moderate to large gastroesophageal varices: non-selective beta-blockers (BBs) and endoscopic variceal ligation (EVL). Meta-analyses show that, compared to no treatment or placebo, both options reduce the risk of first variceal hemorrhage and improve survival in patients with medium/large varices [3]. BBs, have the added benefit of potentially decreasing other complications of portal hypertension (such as ascites) [4], but they have disadvantages including the need for daily medication and adverse events such as fatigue, dizziness and impotence [5]. EVL has the added benefit of visually confirming variceal eradication, but requires conscious sedation, periodic endoscopic surveillance, and is associated with a risk of dysphagia and bleeding ulcers. Combination therapy with both BBs and EVL has not been shown to improve outcomes [6]. Thus, evidence-based guidelines recommend that patients with moderate to large gastroesophageal varices be treated with either BBs or EVL (but not both) to prevent an initial hemorrhage [1,4].

Editorials [7,8] and a previous pilot study [9] demonstrate variability in physician preferences for EVL versus BBs. The reasons underlying this variability, however, have not been studied. The objectives of this study were to examine whether gastroenterologists can be classified into at least two distinct subgroups based on how they prioritize the specific attributes related to BBs and EVL and whether physician characteristics are associated with group membership and treatment preference.

## Methods

Physicians were identified from the American Association for the Study of Liver Diseases and American Gastroenterological Association member directories. For the latter directory, we included only physicians who listed themselves under the “liver”, “clinical practice”, or “esophageal/gastric/duodenal” categories. The resulting lists were classified by state and duplicates were deleted. Email addresses were randomized according to a computer generated random list. Physicians were sent an email containing a link to the online survey, and those that did not respond after a week were contacted by telephone. Individuals who agreed to participate after being telephoned were then sent another email containing a link to take the survey online. The survey began with the following question: “Do you care for patients with cirrhosis?” Only those responding “Yes” were asked to complete the survey.

**Figure 1:**
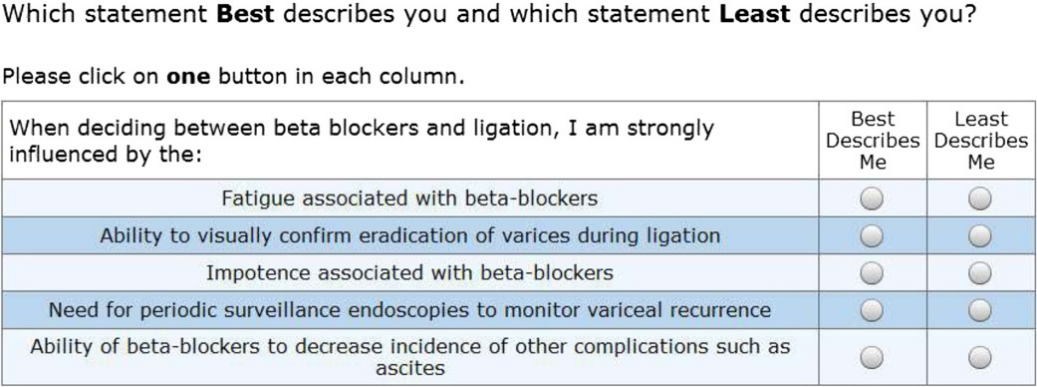
Example of a Best-Worst Question.

We conducted a discrete choice experiment to evaluate how physicians prioritize information when deciding between non-selective BBs and EVL for primary prevention of variceal hemorrhage. Choice format and analyses used Best-Worst scaling (BWS). BWS is a choice task which was developed as an alternative to rating scales in order to obtain respondents’ strength of preferences for a specified set of objects [10]. Surveys using this approach ask respondents to choose the best and worst item from a series of sets containing different combinations of items from a master list (see example in Figure 1). BWS has been recently successfully applied to understand how experts view emerging technologies for hepatocellular carcinoma [11], elicit patients’ preferences for colorectal cancer screening [12], examine barriers to integrating personalized medicine into clinical practice [13], and to develop preference-based quality of life scales [14].

**Figure 2:**
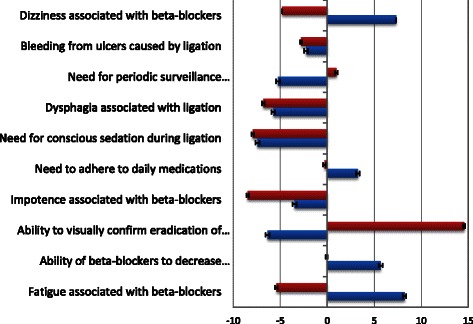
Influence of Treatment Characteristics on Physicians’ Decision Making. Legend: Red bars: Group 1 (n = 80) and Blue bars: Group 2 (n = 30). The scores on the x axis are on a scale from 0–100. Bars to the right of the reference point (=0) represent degree of influence of each attribute. Bars to the left represent the degree to which respondents were not influenced by specific attributes (i.e. attributes which were discarded as being unimportant in their decision making).

Before starting the BWS survey, participants were provided with an example of a BWS question to familiarize them with the method. The survey examined the impact of 10 items, which are listed in Figure 2, on physicians’ decision making. An 11^th^ item “none of these factors influence my decision” was included as a reference category and anchoring point. The balanced list of items (four negative and one positive for each treatment) was developed by a hepatologist who does not perform endoscopy (GGT), a gastroenterologist who does perform endoscopy (LL), a hepatology research fellow (AS), and two investigators with expertise in decision making (LF, JB). For each question, subjects were provided a set of five items and asked to choose the item which had the greatest and least impact on their treatment decision. The experimental design was based on a Youden balanced-incomplete block design [15,16] consisting of 11 choice tasks comparing subsets of five of the objects being examined. Under these experimental conditions, each object appeared five times, and was presented with each of the other 10 objects exactly twice. Unlike other object scaling approaches like using conjoint analysis with a 2^K design, Youden designs offer several important features. First, each choice task has the same number of objects. Next, each item appears the same number of times and, more importantly, it appears with each other factor the same number of times. Finally, as a catalog design, there are a predetermined number of tasks, and each person can receive the same version of the survey.

The BWS choice tasks were followed by a series of questions related to demographic and practice characteristics. Lastly, we asked respondents to indicate their preferred choice of treatment for a standardized patient using the following scenario: “If an upper endoscopy in a 60 year-old man with hepatitis-C cirrhosis (Child-Pugh class B) and no other relevant medical conditions shows large esophageal varices, what is your usual choice for prevention of a first variceal hemorrhage? (Assume the patient has no strong preference for either option)”. Mutually exclusive and exhaustive response options were: 1) Non-selective beta-blockers, 2) Variceal ligation, 3) Both non-selective beta-blockers and variceal ligation, 4) No therapy.

### Statistical analyses

For each respondent, utilities (zero-centered values) were calculated for each level of each attribute using hierarchical Bayes modeling. This approach has the advantage in that it can better incorporate heterogeneity between respondents’ choices [17]. In hierarchical Bayes modeling, the sample averages (prior information) are used to update the individual utilities in a number of iterations until the sample averages stop changing between iterations. After this convergence, the cycle is run several thousand more times and the estimates of each iteration are saved and averaged. We subsequently performed Latent Class analysis to examine whether preferences clustered into at least two segments. Class solutions were replicated five times from random starting seeds.

We examined associations between physician characteristics [training (gastroenterology fellowship vs. hepatology or both), percent of practice devoted to liver disease (<25% vs. ≥ 25%), number of days performing endoscopy (≤1.5 vs. ≥ 2 days), practice setting (academic vs. non-academic), and years in practice (≤20 vs. > 20 years)] with group membership and treatment choice using Chi-square tests. The protocol was approved by the Yale Human Studies Research Program.

## Results

### Physicians’ characteristics

A total of 391 physicians were sent an email over three weeks. Of these, 30 email addresses were invalid and 14 physicians were no longer in practice. Of the remaining 347, 68 reported that they did not care for patients with cirrhosis. Of the 279 physicians who were eligible to participate, 28 completed the survey after receiving the email invitation, and 82 after receiving a phone call. In total, 110 (39%) of eligible physicians completed the survey. Most were over the age of 50 and an overwhelming majority was male. Nearly all had completed fellowships in gastroenterology or both gastroenterology and hepatology and most had spent at least 10 years in practice since completing fellowship. Further details are provided in Table 1.

**Table 1:** **Subject characteristics**

Characteristic	N (Total = 110)
Male	99 (90%)
Age	
<40	27 (25%)
40-50	22 (20%)
51-60	41 (37%)
>60	20 (18%)
Fellowship training	
Gastroenterology	78 (71%)
Hepatology or Liver transplant	1 (1%)
Both	31 (28%)
Years in practice since completing fellowship	
<5	22 (20%)
5-10	11 (10%)
10-20	25 (23%)
>20	52 (47%)
Days per week dedicated to clinical practice	
0	0 (0%)
0.5 (half a day)	3 (3%)
1-3	18 (16%)
>3	89 (81%)
Percent of clinical practice devoted to patients with liver disease	
<25%	65 (59%)
25-50%	25 (23%)
51-75%	7 (6%)
>75%	13 (12%)
Number of patients with cirrhosis seen per month	
0-1	6 (5%)
2-4	22 (20%)
5-10	36 (33%)
11-20	17 (15%)
>20	29 (26%)
Days of endoscopy per week	
0-0.5	8 (7%)
0.5	6 (5%)
1	13 (12%)
2-3	40 (36%)
>3	36 (33%)
Practice setting	
Academic	42 (38%)
Non-academic	68 (62%)
Work at least 25% of time in a non-fee-for-service setting (e.g. Kaiser, VA)	
Yes	14 (13%)
No	96 (87%)

### Physicians’ prioritization of specific treatment attributes

Latent class analysis demonstrated that physicians could be classified into at least two distinct groups (Log-likelihood gain = 404.62 p < 0.001): one larger group composed of 80 members (Group 1) and a smaller group of 30 members (Group 2). The scores demonstrating how strongly physicians’ decisions were impacted by each attribute by group membership are provided in Figure 2. Members of the larger group (Group 1) were influenced primarily by the ability to visually confirm eradications of varices. In contrast, members of Group 2 (n = 30) were influenced primarily by the side effects and mechanism of action of BBs. Forty percent of physicians with practices including 25% or more patients with liver disease belonged to Group 2 compared to 18.5% of physicians with practices having less than 25% patients with liver disease (p = 0.01).

### Predictors of treatment preference

When presented with the standardized scenario describing a 60 year old man with hepatitis C cirrhosis and large esophageal varices, 43% (n = 47) of respondents chose BBs, 18% (n = 20) chose EVL, and 39% (n = 43) chose both. Group membership was associated with treatment choice (Chi-square = 20.9, p = 0.01) (Table 2). Members of Group 1 (i.e. physicians who were influenced primarily by the ability to visually confirm eradications of varices) were more likely to prefer treatment options including EVL (vs. BBs alone) compared to members of Group 2 (46.2% vs. 20.0%, p = 0.01). We found no associations between physicians’ demographic, practice characteristics (including setting), or level of training, and treatment choice.

**Table 2:** **Number (%) of physicians preferring each treatment option according to group membership**

Treatment preferences	Group 1(n = 80)	Group 2 (n = 30)
Beta-Blocker (%)	27 (33.8)	20 (66.7)
Ligation (%)	16 (20.0)	4 (13.3)
Beta-Blocker + Ligation (%)	37 (46.2)	6 (20.0)

## Discussion

Both EVL and BBs are effective in the primary prevention of gastroesophageal variceal bleeding, and guidelines recommend the use of either of these therapies to prevent hemorrhage in patients with cirrhosis with medium/large-sized varices [2,18]. In this study, 39% of the physicians surveyed chose both BBs and EVL as their preferred choice for prevention of first variceal hemorrhage for a 60-year-old man with hepatitis C cirrhosis and large esophageal varices. Thus it appears that combination therapy may be commonly used in clinical practice despite practice guidelines recommending the use of either, but not both options, for primary prophylaxis [7,8].

Preference for using both options may be based on data demonstrating that combined therapy is superior to either monotherapy for prevention of recurrent bleeding from esophageal varices [19]. While combination therapy is recommended [19] for the prevention of recurrent variceal hemorrhage (where the risk of rebleeding is in the order of 60% in one year), a more conservative approach is recommended for primary prophylaxis (where the risk of hemorrhage is ~15% in one year). Failure to adhere to guideline recommendations, however, is well recognized across specialties, and may be due to multiple factors including the level of the guidelines trustworthiness, the perceived relevance of the guideline recommendations to the individual patient, and the strength of the evidence underlying each recommendation [20,21].

The only predictor of treatment preference in this study was the pattern of influence that specific treatment characteristics had on physicians’ decision-making. Those who were influenced primarily by the ability to view eradication of varices were more likely to prefer a prevention strategy including EVL compared to those who were influenced primarily by the treatment characteristics related to BBs. Physician demographic and setting characteristics were not related to their preferred strategy to prevent first variceal bleed.

Most of the physicians in this study (the majority of whom performed endoscopies and had practices with a small number (<25%) of liver patients) were strongly influenced by the ability to visualize the effect of treatment (i.e., eradication of the varices). This observation is consistent with studies demonstrating that physical explanations are significantly more persuasive than less tangible mechanisms of action [22,23]. In contrast, members of the smaller group (Group 2) (60% of whom had practices comprising more than 25% of liver patients) focused primarily on the mechanism of action and side effects of BBs and were more likely to choose BBs alone for primary prophylaxis. Thus the results suggest that members of Group 2 weighed the risk of side effects in their decision making, but preferred the risk-benefit profile of these medications over that of EVL.

The strengths of this study lie in the study design and methods used to evaluate preferences. BWS is a well-validated method of evaluating the influence of specific attributes. Due to an increased awareness of this method in medicine [24,25], this approach has increased in popularity as a measure to inform medical decision making in recent years [12,26,27]. BWS better discriminates between items and between segments of the study population than rating scales; thus, producing more accurate estimates of individual as well as group-level preferences [24,25]. Moreover, scale-related response bias is not a concern with BWS, because respondents make choices instead of indicating their preference using numeric scales. While BWS has many strengths, one of the criticisms of this approach is that it generates priorities on a relative scale. To address this limitation, we included a specific item to serve as a reference category in order to be able to generate scores on an absolute scale.

This study also has certain limitations. While the response rate was modest, it is higher than many surveys involving physicians and the generalizability of our results is constrained by the characteristics of the respondents. The use of standardized scenarios, while enabling us to standardize patients’ clinical characteristics, cannot replicate the complexity of decision making in clinical practice. Because of the extreme variability in cost across insurance plans, this factor was not included in the survey. The relatively small sample size may have limited our ability to find associations between specific practice characteristics and preferences. Almost all participants performed at least some endoscopies, and therefore we could not effectively evaluate this factor as a predictor of decision making or treatment preference. We did not measure access to EVL or physicians’ attitudes towards the clinical practice guidelines, both which may influence treatment preferences. Lastly, we cannot provide data describing non-participants.

## Conclusions

In summary, we found that most of the physicians in this study (the majority of whom performed endoscopies and had practices with a small number (<25%) of liver patients) preferred to a strategy including EVL to prevent first variceal bleed in patients with cirrhosis. This may be due to the strong appeal of being able to visualize eradication of varices. Further research is needed to better understand and confirm this practice pattern and to develop interventions to improve adherence to guidelines.

## Abbreviations

BBs: Beta-blockers
EVL: Endoscopic variceal ligation
n: Number
BWS: Best Worst scaling
